# Application and prospects of AI-based radiomics in ultrasound diagnosis

**DOI:** 10.1186/s42492-023-00147-2

**Published:** 2023-10-13

**Authors:** Haoyan Zhang, Zheling Meng, Jinyu Ru, Yaqing Meng, Kun Wang

**Affiliations:** 1 CAS Key Laboratory of Molecular Imaging, Institute of Automation, Chinese Academy of Sciences, Beijing, 100190 China; 2 School of Artificial Intelligence, University of Chinese Academy of Sciences, Beijing, 100190 China

**Keywords:** Radiomics, Ultrasound imaging, Artificial intelligence, Deep learning, B-mode ultrasound, Color Doppler flow imaging, Ultrasound elastography, Contrast-enhanced ultrasound, Multimodal ultrasound

## Abstract

Artificial intelligence (AI)-based radiomics has attracted considerable research attention in the field of medical imaging, including ultrasound diagnosis. Ultrasound imaging has unique advantages such as high temporal resolution, low cost, and no radiation exposure. This renders it a preferred imaging modality for several clinical scenarios. This review includes a detailed introduction to imaging modalities, including Brightness-mode ultrasound, color Doppler flow imaging, ultrasound elastography, contrast-enhanced ultrasound, and multi-modal fusion analysis. It provides an overview of the current status and prospects of AI-based radiomics in ultrasound diagnosis, highlighting the application of AI-based radiomics to static ultrasound images, dynamic ultrasound videos, and multi-modal ultrasound fusion analysis.

## Introduction

 Ultrasound has become an indispensable tool for medical diagnosis and treatment because of its non-invasiveness, portability, and real-time imaging capabilities [1]. Artificial intelligence (AI)-based radiomics, the use of machine learning algorithms to extract and analyze quantitative features (such as texture, shape, and intensity) from medical images to assist clinicians to diagnose and predict disease prognosis, has attracted research attention in the field of medical imaging, including ultrasound diagnosis [2–4]. It has been extensively studied using various medical imaging modalities, including computed tomography (CT), magnetic resonance imaging (MRI), and positron emission tomography [5–13]. However, compared to other imaging modalities, ultrasound imaging has unique advantages such as high temporal resolution, inexpensiveness, and no radiation exposure. This renders it a preferred imaging modality for several clinical scenarios [14]. The application of AI-based radiomics in ultrasound diagnosis has attracted increasing attention from researchers and clinicians recently.

This review includes a detailed introduction to imaging modalities, including B-mode ultrasound (BUS) [15], color Doppler flow imaging (CDFI) [16], ultrasound elastography (UE) [17], contrast-enhanced ultrasound (CEUS) [18], and multi-modal fusion analysis of BUS, CDFI, UE, and CEUS. It aims to provide an overview of the current status and prospects of AI-based radiomics in ultrasound diagnosis by highlighting the application of AI-based radiomics to static ultrasound images, dynamic ultrasound videos, multi-modal ultrasound fusion analysis, and advanced methods of intelligent ultrasound analysis.

AI has three fundamental tasks in the field of ultrasound: classification, segmentation, and detection. Static ultrasound involves two-dimensional imaging. Furthermore, BUS images reflect the anatomical structure of lesions and are commonly used for classification and segmentation tasks. Additionally, CDFI images reflect the blood flow information of lesions, whereas UE images reflect the tissue hardness of lesions. These two modalities are often used for classification tasks. Dynamic ultrasound involving videos (BUS videos) dynamically reflect changes in the anatomical structure and position of lesions and are frequently used for tasks such as classification, segmentation, detection, and tracking. In addition, CEUS reflects dynamic changes in blood flow and is commonly used for classification. The relationships between AI tasks and different ultrasound modalities are shown in Table 1.

**Table 1:** Relevance of AI tasks and different modalities

Static ultrasound			Dynamic ultrasound	
**BUS (image)**	**CDFI**	**UE**	**BUS (video)**	**CEUS**
Classification	Classification	Classification	Classification	Classification
			Segmentation	
Segmentation			Lesion detection	
			Lesion tracking	

BUS images are commonly used for classification and segmentation tasks. Additionally, CDFI and UE images are often used for classification tasks. Dynamic ultrasound involving videos (BUS videos) are frequently used for tasks such as classification, segmentation, detection, and tracking. In addition, CEUS videos are commonly used for classification

In the next few sections, the applications of AI in ultrasound diagnosis will be discussed in detail. In the static ultrasound image section, the application of AI-based radiomics to extract quantitative features (such as tumor size, shape, texture, and echogenicity) from ultrasound images to assist in the diagnosis and differentiation of various diseases is discussed. The application of AI-based radiomics to analyze changes in the intensity and texture of lesions during the CEUS process is introduced in the dynamic ultrasound video section. This can provide valuable information for the diagnosis and treatment of diseases.

In the multi-modal ultrasound fusion analysis section, the use of AI-based radiomics to integrate information from multiple ultrasound modalities, such as BUS, CDFI, UE, and CEUS, and improve the accuracy of disease diagnosis and treatment planning is discussed. Tables 2, 3 and 4 present the related studies, their aims, and the performance of the AI algorithms, respectively. In addition, the challenges and limitations of AI-based radiomics in ultrasound diagnosis, such as the lack of standardization in feature extraction and the need for large-scale multicenter studies to validate the clinical efficacy of AI-based radiomics is discussed.

**Table 2:** The existing research in the area of static ultrasound

Reference	Number of patient	Tumor characteristic	Imaging modality	Function and prediction result	Method
Ye et al. [19]	1844 images	Triple negative breast cancer	BUS	Benign vs TN (AUC): 0.9789, benign vs NTN (AUC): 0.9689, TN vs NTN (AUC): 0.9000	Resnet50
Zhou et al. [20]	192	Axillary lymph node metastasis	BUS	Predicting ALN metastasis, AUC = 0.85	LASSO
Kwon et al. [21]	169	Distant metastasis of follicular thyroid carcinoma	BUS	Distant metastasis classification, AUC = 0.90	SVM
Meshram et al. [22]	101	Carotid plaque	BUS	Dice coefficients for automatic is 0.55, for semi-automatic is 0.84	Dilated U-Net
Wang et al. [23]	398	Liver fibrosis	UE	Diagnosing liver fibrosis stages AUC(F4) = 0.97, AUC (≥ F3) = 0.98, AUC (≥ F2) = 0.85	CNN
Tahmasebi et al. [24]	381	Axillary lymph nodes	UE	Classification of axillary lymph nodes, AuPRC = 0.78	Google cloud autoML vision, mountain view
Lu et al. [25]	807	Liver fibrosis	UE	Discrimination of significant fibrosis, AUC = 0.91	CNN
Zhou et al. [26]	297	Liver fibrosis	UE	Assess liver fibrosis stages, AUC (cirrhosis and advanced fibrosis) = 0.98, AUC (significance fibrosis) = 0.76	CNN

**Table 3:** The existing researches in the area of dynamic ultrasound

Reference	Number of patient	Tumor characteristic	Imaging modality	Function and prediction result	Method
Tong et al. [27]	558	Pancreatic ductal adenocarcinoma, chronic pancreatitis	CEUS	AUC in internal validation is 0.978 (95%CI: 0.950–0.996)	Deep learning radiomics (DLR) model
Chen et al. [28]	221	Breast cancer	CEUS	Sensitivity of 97.2% and an accuracy of 86.3%	Three-dimensional convolutional neural network (CNN) model
Liu et al. [29]	130	HCC	CEUS	AUC (R-DLCEUS) = 0.93, AUC (radiomics-based time intensity curve of the CEUS model (R-TIC)) = 0.80, AUC (radiomics-based BUS image model (R-BMode)) = 0.81	R-DLCEUS, R-TIC, R-BMode
Liu et al. [30]	419	Very-early or early stage HCC	CEUS	17.3% RFA patients and 27.3% SR patients should swap their treatment	Deep learning-based radiomics model
Sun and Lu [31]	156	Diabetic nephropathy	CEUS	Experimental group kidney volume: 136.07 ± 22.16 cm^3^, control group kidney volume: 159.11 ± 31.79 cm^3^	Poisson three-dimensional reconstruction algorithm
Meng et al. [32]	CMLN dataset: 199, BL dataset: 146	Metastasis cervical lymph nodes, breast lesion	CEUS	CMLN: 91.05% dice and 80.06% IOU; BL: 89.97% dice and 75.62% IOU	CEUSegNet
Iwasa et al. [33]	100	Pancreatic tumors	CEUS	IOU: 0.77	U-Net

**Table 4:** The existing researches in the area of dual-/multi-modal ultrasound fusion analysis

Research area	Modality	Method	Objective	Performance
Clinical application research	BUS, UE	Deep polynomial network [34]	Differentiating malignant and benign breast tumors	AUC: 0.961
	BUS, UE	Nomogram [35]	Prediction of malignant status of breast lesions	AUC: 0.920
	BUS, UE	Deep learning [36]	Differentiating malignant and benign breast tumors	Accuracy: more than 90%
	BUS, CDFI, UE	Deep learning [37]	Assessment of breast cancer risk	AUC: 0.922 for dual-modal and 0.955 for tri-modal method
	BUS, UE	LASSO [38]	Differentiating benign, lymphomatous, and metastatic lymph nodes	AUC: 0.960 for benign vs lymphomatous, 0.716 for benign vs metastatic, 0.933 for lymphomatous vs metastatic, and 0.856 for benign vs malignant
	BUS, UE	Scoring and support vector machine (SVM) [39]	Evaluation of axillary lymph node metastasis in breast cancer patients	AUC: 0.881 for scoring and 0.895 for SVM
	BUS, CDFI	Deep learning [40]	Diagnosis of unexplained cervical lymphadenopathy	AUC: 0.873, 0.837, and 0.840 in the three testing cohorts for four common etiologies
	BUS, UE	Deep learning [41]	Grading liver fibrosis	AUC: 0.950, 0.932, and 0.930 for classifying S4, ≥ S3, and ≥ S2
	BUS, UE and shear wave viscosity imaging	Sparse representation theory and SVM [42]	Diagnosis and clinical prediction of HCC	AUC: 0.94 for benign and malignant classification, 0.97 for malignant subtyping, 0.97 for PD-1 prediction, 0.94 for Ki-67 prediction, and 0.98 for MVI prediction
	BUS, CDFI, UE	Deep learning [43]	Diagnosis of suspicious thyroid nodules	AUC: 0.928
	BUS and superb microvascular imaging ultrasound	SVM [44]	Differentiation between gallbladder neoplastic polyps and cholesterol polyps	AUC: 0.850
	BUS, UE, CEUS	Logistic regression and nomogram [45]	Prediction of microvascular invasion and recurrence of HCC	AUC: 0.789
Algorithm research	BUS, CDFI, UE	A self-supervised multi-modal fusion network [46]	Diagnosing thyroid nodules	Accuracy: 89.79%, higher than other deep learning methods
	BUS, CDFI, shear-wave and strain UE	An modality auto-weighting and recovery framework [47]	Diagnosing breast cancer	Accuracy: 92.63% for modality completeness and 90.65% for modality missing
	BUS, CDFI, UE and CEUS	Multi-step modality fusion network [48]	Identifying the histologic subtypes of metastatic cervical lymphadenopathy	Accuracy: 80.06%, true-positive rate: 81.81%, and true-negative rate: 80.00%
	BUS, CDFI	Tissue-aware cervical lymph node diagnosis method via multi-modal ultrasound semantic segmentation [49]	Post-pandemic healthcare for COVID-19 vaccine	Accuracy: 82.54%
	BUS, CEUS	Cross-modality lesion segmentation network [32]	Semantic segmentation	IOU: 80.06% for metastasis cervical lymph nodes and 75.62% for breast lesions

AI-based radiomics has the potential to revolutionize the field of ultrasound diagnosis by providing objective and quantitative analyses of ultrasound images. This can improve the accuracy and efficiency of disease diagnosis and treatment. This review provides valuable insights into the current status and future prospects of AI-based radiomics in ultrasound diagnosis and serves as a useful reference for researchers and clinicians in this field.

## Application of AI-based radiomics in ultrasound diagnosis

### Static ultrasound

**Fig. 1:**
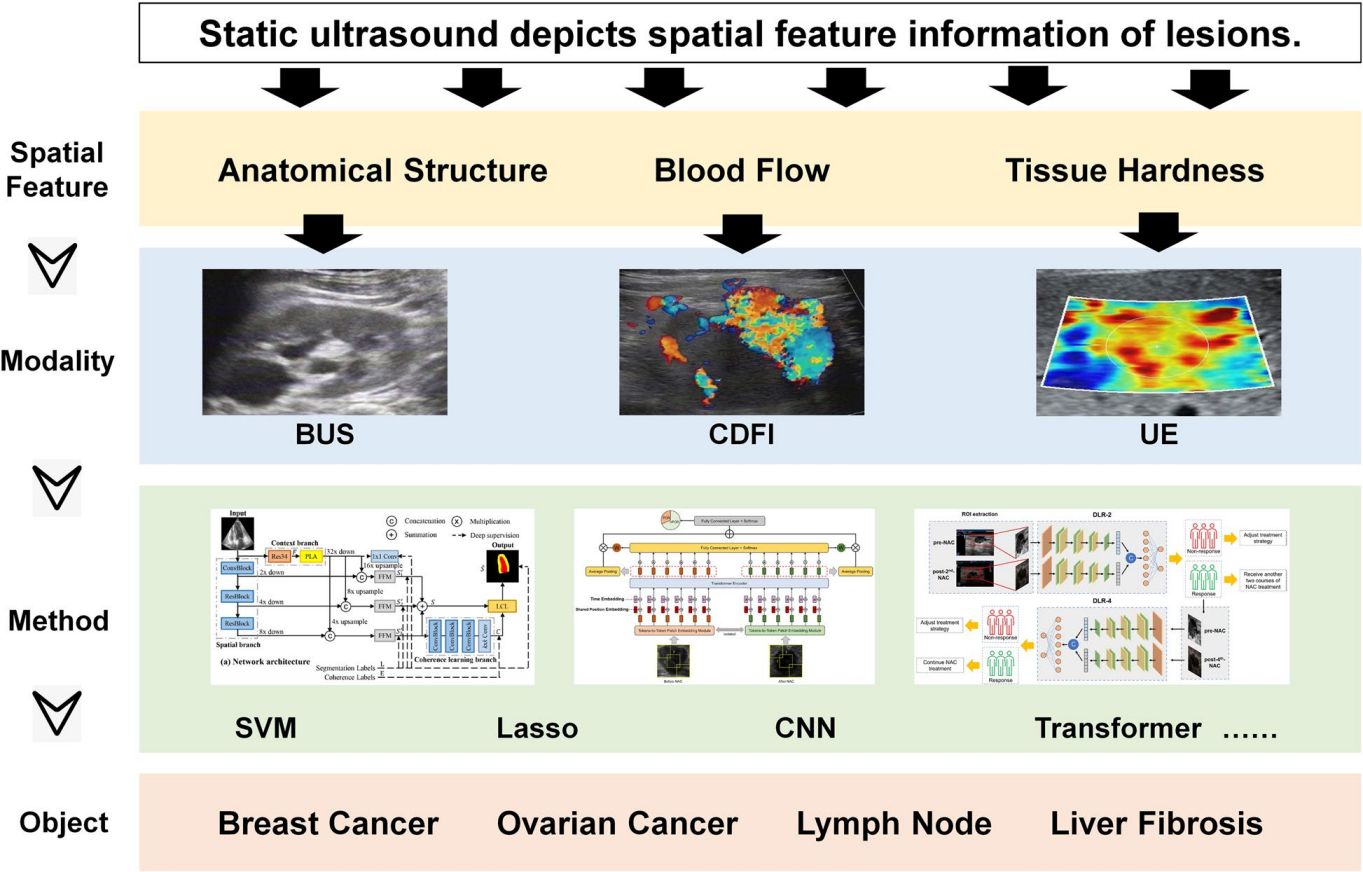
Static ultrasound analysis model for diagnosis and prediction. Static ultrasound reflects the spatial characteristics of lesions. It involves two-dimensional imaging. The BUS images provide information on the anatomical structure, shape, texture, and position of lesions. Moreover, the CDFI images reveal the direction of blood flow in the lesions. Additionally, UE images reflect the tissue hardness of the lesions. The existing intelligent analysis methods for static ultrasound include SVM, lasso regression, CNNs, and transformers. These are commonly used for the diagnosis and prognosis of diseases such as breast cancer, ovarian cancer, lymph nodes, and liver fibrosis

Static ultrasound refers to the use of BUS, color CDFI, and UE to generate static images of tissues and organs within the body. These modalities are widely used in medical diagnosis and research, and they provide valuable information on the morphology, vascularity, and elasticity of various tissues. Static ultrasound imaging modalities provide detailed spatial information about tissues and organs, rendering them valuable tools for medical diagnosis and research. Owing to the advancement in technology and an increased understanding of its clinical utility, static ultrasound plays an important role in AI-based radiomics in ultrasound diagnosis (Fig. 1).

#### BUS

This is the most common type of ultrasonography and involves various examinations. Analysis of ultrasound images performed using AI differs for different examination sites and purposes. It mainly performs classification, segmentation, and detection tasks.

The most common classification task is diagnostics. Specifically, there are diagnoses of specific diseases by traditional and deep learning, such as diagnosis of hepatocellular carcinoma (HCC) [50], triple negative breast cancer [19], cervical tuberculous lymphadenitis [51], and v-raf murine sarcoma viral oncogene homolog B (BRAF) mutation in thyroid cancer [52]. These are generally dichotomous tasks (diagnosis as positive or negative) and staging of the same lesion (such as liver fibrosis staging [41], lung B-line severity assessment [53], classification of COVID-19 markers [54], and determination of the type of liver lesion [55]). They are generally multi-classified tasks. Additional categorical tasks include the preoperative prediction of Ki-67 expression levels in breast cancer patients [56], prediction of axillary lymph node metastasis in breast cancer patients [20], and distant metastasis in follicular thyroid cancer [21]. The classification task usually extracts the features of BUS images from the region of interest (ROI) selected by the doctor. Some of them add relevant data features and use them to make judgments. Some of the tasks generate heat maps by the AI after the judgment, which explains the AI’s judgment and inspires the doctors to summarize their experiences. A heatmap is an image that describes which regions of an input image a model focuses on. The colors’ intensity in the heatmap indicates the level of attention the model gives to corresponding areas. Doctors can use heatmaps to understand which regions the model prioritizes. This enables targeted observation and study of those specific areas, leading to the accumulation of experience for diagnosis and prognosis prediction.

The classification task makes a judgment on the entire image, whereas the detection task adds a localization task to the judgment. This can localize the carotid artery cross-section in ultrasound images [57], lesions on abdominal ultrasound images [58], and thyroid nodules [59]. The detection task can generate multiple labels on a single BUS image. It uses rectangular boxes to mark the positions corresponding to the labels and can handle multiple ROIs in a single image.

The segmentation task is a more accurate classification task. It classifies each pixel point, after which information such as the boundary, size, and location of different regions in the ultrasound image becomes clear. AI can perform arterial plaque [22, 60, 61], breast tumor [62, 63], and thyroid nodule [64] segmentations on ultrasound images. Segmentation can precisely show the edge shape of an ROI, and this study is mainly based on segmentation. After segmentation, AI can continue with measurement and estimation tasks such as the measurement of forced muscle thickness [65], measurement of total carotid plaque area [66, 67], high-risk plaque detection [68], CIMT estimation [69, 70], and estimation of skeletal muscle status [71].

Additionally, for a continuous two-dimensional BUS, AI can perform three-dimensional reconstruction [72, 73], motion tracking [74, 75], motion estimation [76], and other three-dimensional analyses. Moreover, AI accomplishes the above functions by autonomously synthesizing ultrasound datasets [77] with results comparable to real data. This solves the problems of dataset shortage and patient privacy.

The integration of AI into BUS (ultrasound) has led to significant advancements in medical diagnosis and analyses. Additionally, AI + BUS can perform various tasks such as classification, detection, and segmentation. This enables the accurate diagnosis of specific diseases such as HCC, breast cancer, and thyroid nodules. It can also aid in predictive tasks such as estimating expression levels, lymph node metastasis, and distant metastasis. This transformative technology has valuable medical applications.

#### CDFI

This is another commonly used technique in medical ultrasonography that provides information on blood flow. It uses color to indicate the direction and velocity of blood flow in real time [78]. Combining BUS with CDFI can provide valuable diagnostic information regarding blood flow in the imaged structures. This can be particularly useful in diagnosing conditions such as deep vein thrombosis, peripheral arterial disease, and other vascular abnormalities.

The applications of BUS and CDFI focus on the classification of breast cancer molecular subtypes. Yu et al. [79] proposed a CNN for the classification of breast masses (including inflammatory masses, adenosis, benign tumors, and malignant tumors) using BUS and CDFI images. This study includes 3623 patients. The AUCs for the classification of benign and malignant tumors, inflammatory masses, and adenosis were 0.90, 0.91, 0.90, and 0.89, respectively. The results showed that the CNN model outperformed human radiologists and had high accuracy. This suggests that it can be a valuable tool for clinical practice. Shen et al. [80] proposed an AI system that achieved radiologist-level accuracy in identifying breast cancer in BUS and CDFI images. Furthermore, this study addresses the high false-positive rate of breast ultrasound. The AI system was developed on a dataset of 288767 examinations, comprising 5442907 BUS and CDFI images. In a retrospective reader study, the AI achieved a higher AUROC than the average of ten board-certified breast radiologists (AUROC: 0.962 AI, 0.924 ± 0.02 radiologists). The use of the AI system also resulted in a 37.3% decrease in false-positive rates and a 27.8% reduction in the number of requested biopsies, while maintaining the same level of sensitivity. Sultan et al. [81] proposed the use of machine learning with multi-modal ultrasound (including BUS and CDFI) to enhance the diagnosis of breast cancer. The BUS and CDFI results for 160 biopsy-proven breast lesions were quantitatively analyzed. The AUC for the BUS features was 0.85, whereas that for the combined BUS and CDFI was 0.89. After pruning the high-dispersion cases, AUC improved to 0.96. This study demonstrates the effectiveness of machine learning with multi-modal ultrasound in achieving high diagnostic performance for breast cancer diagnosis. The use of dispersion to identify weakly learned cases can markedly enhance diagnosis. However, further studies are required to validate these results in larger and more diverse patient populations.

BUS and CDFI are also widely used to diagnose other diseases. Wang et al. [82] proposed a CNN model trained on the Keras machine learning platform with a modified pre-trained VGG16 network to classify benign and malignant soft tissue masses on ultrasound images and to differentiate between three commonly observed benign masses. The dataset used in the study included BUS and CDFI images from 419 patients, with 227 patients having a histologic diagnosis confirmed by biopsy or surgical excision and 192 patients with masses that demonstrated imaging characteristics of lipoma, benign peripheral nerve sheath tumor, and vascular malformation. The CNN model, trained to classify malignant and benign masses, achieved an accuracy of 79% on the test data. This is consistent with the performance of two experienced musculoskeletal radiologists. The area under the curve for the model was 0.91. The model trained to differentiate among the three benign masses achieved an accuracy of 71% for the test data [82]. Wu et al. [83] proposed a deep multi-modal learning network for the prediction of lymph node metastasis in patients with primary thyroid cancer. The network integrated the clinical context from health records and various imaging modalities. The dataset used in this study comprises 1536 patients’ BUS, CDFI, and clinical records. The proposed deep multi-modal learning network achieved an average F1 score of 0.888 and AUC value of 0.973 in two independent validation sets. This performance is significantly better than that of the three single-modality deep learning networks. The proposed MMC-Net model achieves significant improvements over the single-modality networks. Moreover, the proposed MCI index provides insight into the contribution levels of different modalities, which can enhance radiologists’ understanding of the model’s predictions. However, this study had some limitations, such as the relatively small size of the multi-modal dataset and lack of data from other hospitals. Therefore, further studies using larger and more diverse datasets are recommended.

The CDFI is rarely used for only intelligent analysis; it is typically combined with a BUS. The combination of BUS and CDFI in medical ultrasound has shown promising results, particularly in the diagnosis of molecular subtypes of breast cancer and other diseases. Machine learning with BUS and CDFI demonstrates the potential of AI for improving diagnostic accuracy and patient care.

#### UE

AI has resulted in the emergence of the application of UE measurements, thereby offering a partial solution to the limitations of UE. This can enhance the diagnostic accuracy of this technique and facilitate its seamless integration into routine clinical practice.

Moreover, UE-based AI can assist in quantitative ultrasound imaging data analysis and integration for the assessment of liver diseases with the aim of developing individualized classifications and predictive models. Wang et al. [23] proposed the use of a newly developed deep learning radiomics of elastography (DLRE) model to assess liver fibrosis stages. DLRE adopts a radiomic strategy for the quantitative analysis of heterogeneity in two-dimensional shear wave elastography (SWE) images. This study aimed to address the clinical problem of accurately diagnosing the stages of liver fibrosis in hepatitis B virus-infected patients using noninvasive methods, a significant challenge for conventional approaches. Zhou et al. [26] proposed deep learning radiomics of an elastography model, which adopted a CNN based on transfer learning as a noninvasive method to assess liver fibrosis stages. This is essential for the prognosis and surveillance of chronic hepatitis B patients. Lu et al. [25] proposed an updated deep-learning radiomics model of elastography (DLRE2.0) to discriminate significant fibrosis (≥ F2) in patients with chronic liver disease. The dataset used in their study included 807 patients and 4842 images from three hospitals. The DLRE2.0 model showed a significantly improved performance compared to the previous DLRE model with an AUC of 0.91 for evaluating ≥ F2. The radiomics models showed good robustness in an independent external test cohort. Destrempes et al. [84] proposed a machine learning model based on random forests to select combinations of quantitative ultrasound features and SWE stiffness for the classification of steatosis grade, inflammation grade, and fibrosis stage in patients with chronic liver disease.

The UE-based AI can also improve the classification and diagnosis of lymph diseases. Zhang et al. [85] used a random forest model for the differential diagnosis of thyroid nodules based on conventional ultrasound and real-time UE. Qin et al. [86] proposed a method based on a CNN that combined the characteristics of conventional ultrasound and ultrasound elasticity images to form a hybrid feature space for the classification of benign and malignant thyroid nodules. Zhao et al. [87] used a machine learning model that incorporated radiomic features extracted from ultrasound and SWE images to develop ML-assisted radiomics approaches. Liu et al. [88] proposed a radiomic approach using BUS and strain elastography to estimate the lymph node status in patients with papillary thyroid carcinoma.

UE is an adjunctive instrument for refining the identification and non-intrusive delineation of breast lesions, facilitating radiologists in amplifying patient care. The incorporation of SWE into BUS demonstrated the potential to provide supplementary diagnostic insights and to reduce the need for unwarranted biopsies [89]. Li et al. [90] proposed an innovative dual-mode AI architecture that could automatically integrate information from ultrasonography and SWE to assist in breast tumor classification. Kim et al. [91] used deep learning-based computer-aided diagnosis and SWE with BUS to evaluate breast masses detected by screening ultrasound and addressed the low specificity and operator dependency of breast ultrasound screening.

Similarly, UE is also widely used to diagnose other diseases. Additionally, UE-based AI can improve lymph node classification, compared to that performed only by radiologist evaluations. Tahmasebi et al. [24] evaluated an AI system for the classification of axillary lymph nodes on UE. They aimed to compare the performance of the AI system with that of experienced radiologists in predicting the presence of metastasis to the axillary lymph nodes on ultrasound.

AI + UE has shown considerable potential in revolutionizing medical diagnoses. It offers a partial solution to the limitations of UE, enhances diagnostic accuracy, and enables individualized classification and predictive models for liver diseases. Additionally, UE-based AI can aid in lymph node disease classification and non-intrusive delineation of breast lesions, reducing the need for unnecessary biopsies and improving patient care. Furthermore, the application of AI to other diseases (such as lymph node classification) highlights its ability to outperform radiologist evaluations. The combination of AI and UE can advance medical imaging and patient outcomes.

#### Waveform graph

Doppler ultrasound is capable of measuring blood flow velocity, which results in a waveform graph showing the blood supply. No prior studies were found for the use of AI to analyze Doppler flow spectrograms. However, a study conducted to diagnose early allograft dysfunction in liver transplant patients showed that a deep learning model could be used to analyze and determine blood flow spectrograms. The results of the AI analysis of the flow velocity spectrograms related to blood flow can lead to medical conclusions related to blood flow, such as determining the cause of the disease to assist the physician’s treatment. Because the results learned by AI are significantly better than the empirical results of doctors, the heat maps generated assist doctors to understand the information contained in blood flow spectrograms.

### Dynamic ultrasound

**Fig. 2:**
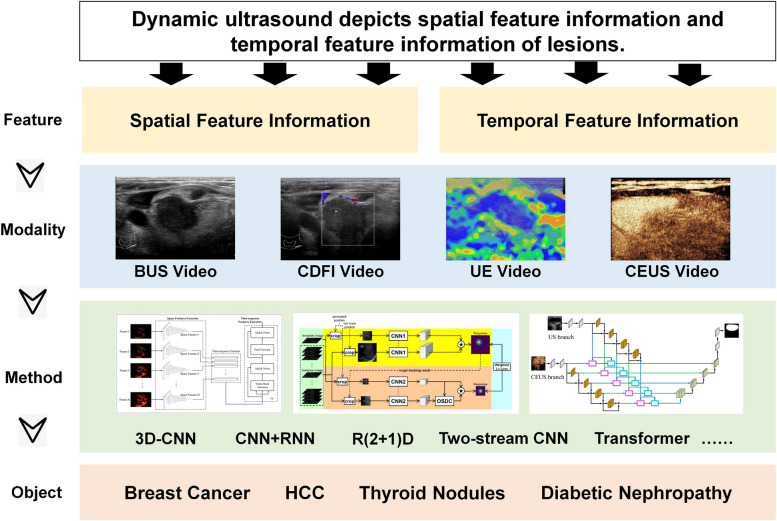
Dynamic ultrasound analysis model for diagnosis and prediction. Dynamic ultrasound reflects the spatiotemporal characteristics of lesions. It involves videos. Common ultrasound modalities include BUS, CDFI, UE, and CEUS. Current studies on ultrasound videos primarily focus on BUS and CEUS videos. The commonly used intelligent analysis methods for ultrasound videos include three-dimensional CNN, CNN + RNN, R(2 + 1)D, dual-stream CNN, and transformers. These are frequently employed for the diagnosis and prognosis of diseases such as breast cancer, liver cancer, thyroid nodules, kidney disease, and diabetes

Dynamic ultrasound (also known as CEUS) is an effective imaging tool for analyzing the spatiotemporal characteristics of lesions and diagnosing or predicting diseases. Real-time high-resolution images produced by CEUS are often comparable to those obtained by CT or MRI [92]. Thus, a quick, reliable, and relatively inexpensive CEUS may reduce the need for additional testing. Additionally, CEUS is routinely used worldwide to detect heart disease and stratify the risk of heart attack or stroke. It is also used to identify, characterize, and stage tumors of the liver [93], kidney [94], prostate [95], breast [96], and other organ systems and to monitor the effectiveness of cancer therapies. However, because CEUS is considerably affected by operator bias, the image quality is relatively unstable and the tumor boundary (TB) is often unclear. These disadvantages often limit the accuracy of the direct analysis by radiologists. Therefore, several studies recently tend to use deep learning methods to extract spatiotemporal features from CEUS sequences to assist treatment (Fig. 2).

Furthermore, AI deep-learning models can aid in diagnostic classification. Tong et al. [27] developed an end-to-end DLR model based on CEUS to assist radiologists in identifying pancreatic ductal adenocarcinoma and chronic pancreatitis. To train and test the model, 558 patients with pancreatic lesions were included. The DLR model achieved an AUC of 0.986 (95%CI: 0.975–0.994), 0.978 (95%CI: 0.950–0.996), 0.967 (95%CI: 0.917–1.000), and 0.953 (95%CI: 0.877–1.000) in the training, internal validation, and external validation cohorts 1 and 2, respectively. Similarly, Chen et al. [28] proposed a three-dimensional CNN model based on CEUS videos. This model was validated using a Breast-CEUS dataset comprising 221 cases. The results show that the proposed model in this study achieved a sensitivity and an accuracy of 97.2% and 86.3%, respectively. The incorporation of domain knowledge led to a 3.5% and 6.0% improvement in sensitivity and specificity, respectively. Liu et al. [29] established and validated an AI-based radiomics strategy for predicting personalized responses of patients with HCC to the first transarterial chemoembolization (TACE) session by quantitatively analyzing CEUS cines. One hundred and thirty patients with HCC (89 and 41 for training and validation, respectively) who underwent ultrasound examinations (CEUS and BUS) within one week before the first TACE session were retrospectively enrolled. The AUCs of deep learning radiomics-based CEUS model (R-DLCEUS), machine learning R-TIC, and machine learning radiomics-based BUS images model were 0.93 (95%CI: 0.80–0.98), 0.80 (95% CI:, 0.64–0.90), and 0.81 (95%CI: 0.67–0.95) in the validation cohort, respectively.

In addition, AI deep learning algorithms can be used to select and predict treatment methods and effects, respectively. Liu et al. evaluated the performance of a deep learning-based radiomics strategy designed for analyzing CEUS to predict the progression-free survival (PFS) of radiofrequency ablation (RFA) and surgical resection (SR) and to optimize the treatment selection for patients with very early or early stage HCC [30]. Their study retrospectively enrolled 419 patients examined using CEUS within one week before receiving RFA or SR (RFA: 214, SR: 205) between January 2008 and 2016. The R-RFA and SR showed remarkable discrimination (C-index: 0.726 and 0.741 for RFA and SR, respectively). The model identified that 17.3% and 27.3% of the RFA and SR patients should swap their treatment, indicating that their average probability of two-year PFS would increase by 12% and 15%, respectively. Similarly, Sun and Lu [31] evaluated the efficacy of atorvastatin in the treatment of diabetic patients using CEUS, based on a three-dimensional reconstruction algorithm. One hundred and fifty-six DN patients were divided into experimental (conventional treatment + atorvastatin) and control (conventional treatment) groups. The kidney volume and hemodynamic parameters, including the maximal kidney volume, minimal kidney volume, and resistance index of all patients were measured and recorded before and after treatment. The volume (136.07 ± 22.16 cm^3^) in the experimental group after the treatment was smaller, in contrast to the control group (159.11 ± 31.79 cm^3^) (*P* < 0.05).

Because delineating the ROI containing the lesion and the surrounding microvasculature frame-by-frame in CEUS is a time-consuming task [32], some AI methods have been proposed to realize automatic segmentation of lesions. Meng et al. [32] proposed a novel U-net-like network with dual top-down branches and residual connections known as CEUSegNet. The CEUSegNet uses the US and CEUS parts of a dual-amplitude CEUS image as inputs. The lesion position can be determined exactly under US guidance, and the ROI can be delineated in the CEUS image. Regarding the CMLN dataset, CEUSegNet achieved 91.05% Dice and 80.06% intersection over the union (IOU). Considering the BL dataset, CEUSegNet achieved 89.97% Dice and 75.62% IOU. Iwasa et al. [33] evaluated the capability of the deep learning model U-Net for automatic segmentation of pancreatic tumors on CEUS video images and the possible factors affecting automatic segmentation. This retrospective study included 100 patients who underwent CEUS for pancreatic tumors. The degree of respiratory movement and TB were divided into three-degree intervals for each patient and were evaluated as possible factors affecting the segmentation. The concordance rate was calculated using IOU. The median IOU for all the cases was 0.77. The median IOU for TB-1 (approximately clear), TB-2, and TB-3 (more than half unclear) were 0.80, 0.76, and 0.69, respectively. The IOU of TB-1 was significantly higher than that of TB-3 (*P* < 0.01).

Thus, CEUS is a powerful tool for diagnosing and predicting various diseases. It offers real-time high-resolution images comparable to those of CT or MRI, thereby reducing the need for additional tests. Although AI-based deep learning models are affected by operator variability and unclear tumor boundaries, they can assist radiologists in obtaining precise diagnosis and treatment selection. Furthermore, AI models have achieved impressive results in classifying pancreatic lesions, predicting liver fibrosis stages, and optimizing the treatment choices for patients with HCC. Additionally, AI can aid in lesion segmentation, automate time-consuming processes, and improve accuracy. Integrating AI with CEUS has immense potential for advancing medical imaging and improving patient outcomes.

### Dual-/multi-modal ultrasound and AI-powered ultrasound image analysis

**Fig. 3:**
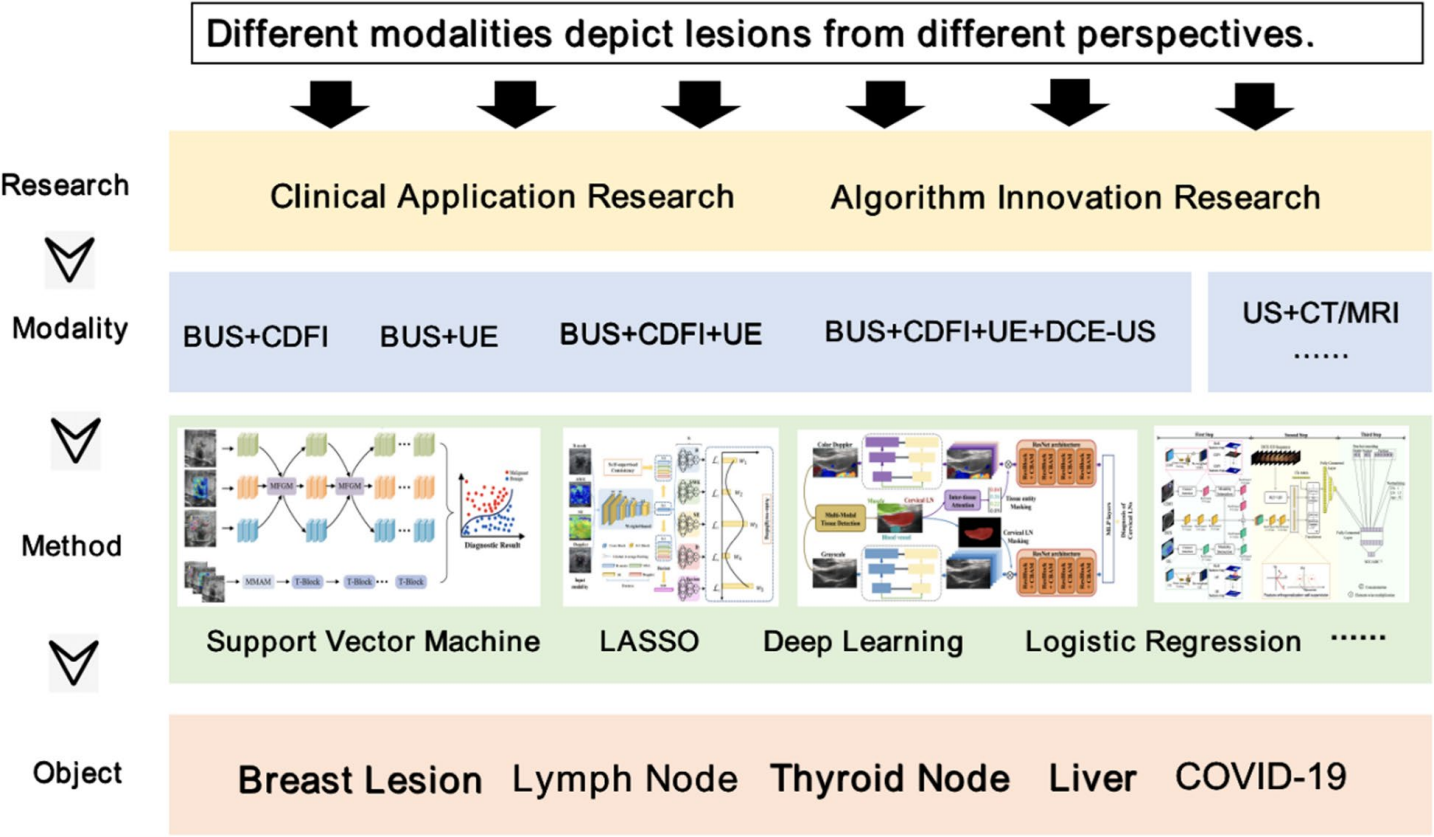
Research ideas for dual-/multi-modal ultrasound fusion analysis. The fusion analysis of different ultrasound modalities is of significant clinical importance. Each modality of ultrasound has its own advantages and limitations, and an efficient modality fusion method can complement each modality. This improves the accuracy of ultrasound AI diagnosis. Currently, there are various modality combinations such as BUS + CDFI, BUS + UE, BUS + CDFI + UE + CEUS, and US + CT/MRI. Common fusion methods include logistic regression, machine, and deep learning. These are frequently used to diagnose diseases such as breast cancer, liver cancer, thyroid nodules, and COVID-19

Various ultrasound modalities depict lesions from different aspects, which provides clinicians with the power to understand lesions more comprehensively. Naturally, a more satisfactory performance is expected via intelligent fusion analysis of combinations of ultrasound modalities. Existing studies in the area of dual/multi-modal ultrasound fusion analysis can be divided into two parts: clinical application and algorithm studies, which focus on the performance of specific clinical problems and the development of fusion methods. Figure 3 summarizes the related studies in recent years.

Regarding clinical application studies, two or three performance-based ultrasound modalities have attracted research attention with the former occupying a larger proportion. As a common disease, breast-related diseases have received widespread attention. Using the same ultrasound modality combinations, Zhang et al. [34] compared the performance of single- and dual-modal methods based on principal component analysis, multiple kernel learning, and deep polynomial network for the classification of breast tumors and achieved an AUC of 0.961. To predict the malignant status of breast lesions, Jiang et al. [35] developed a nomogram to extract and analyze BUS and UE features and achieved an AUC of 0.92, which was close to the level of radiologists. In contrast to these methods, deep learning methods were proposed by Misra et al. [36] to classify a breast lesion as benign or malignant and achieved an accuracy of more than 90%, which was better than that of individual models trained using only BUS or SE images. Qian et al. [37] proposed an explainable deep learning system for the assessment of breast cancer risk and evaluated lesions with an AUC of 0.955 based on BUS, CDFI, and UE. The lymph nodes are another concern. Based on BUS and UE, Chen et al. [38] used a machine learning method to differentiate between benign, lymphomatous, and metastatic lymph nodes and achieved an AUC of 0.856 for the classification of benign and malignant lymph nodes. To evaluate axillary lymph node metastasis in patients with breast cancer, Zhang et al. [39] used scoring and SVM approaches to analyze BUS and UE manual features and obtained a maximum AUC of 0.895. Zhu et al. [40] developed an ensemble deep learning model for the diagnosis of four common unexplained cervical lymphadenopathy types and implemented two-stage, multi-center, and multi-year experiments. The results showed that it achieved an accurate diagnosis of unexplained CLA and narrowed the gap between radiologists with different levels of experience. This can potentially benefit patients with CLA in underdeveloped countries and regions worldwide. Related studies have also explored the diagnostic ability of machine learning methods for other diseases. For example, Xue et al. [41] graded liver fibrosis using the Inception-V3 network and obtained AUCs higher than 0.9 for each class. Yao et al. [42] developed five radiomics models for the diagnosis and clinical behavior prediction of HCC using BUS, UE, and shear wave viscosity imaging. This aided in comprehensive liver tumor evaluations, including diagnosis, differential diagnosis, and clinical prognosis. Tao et al. [43] employed deep-learning models based on the BUS, CDFI, and UE. They showed excellent performance in the differential diagnosis of suspicious thyroid nodules, increased the diagnostic efficacy of thyroid nodule evaluations by junior radiologists, and provided an objective assessment of the clinical and surgical management phases that followed. In contrast, BUS and superb microvascular imaging ultrasound were adopted in ref. [44] to improve the accuracy of differentiation between gallbladder neoplastic polyps and cholesterol polyps. To predict microvascular invasion and HCC recurrence, Zhong et al. [45] developed a nomogram based on BUS, UE, and CEUS manual features and provided an effective tool to stratify the risk of recurrence and guide individualized treatment of HCC.

Researchers have conducted certain studies in the field of algorithms to explore the optimal method for multimodal ultrasound fusion analysis. Xiang et al. [46] proposed a self-supervised multimodal ultrasound fusion network and suggested a multimodal–multihead attention branch and multimodal feature guidance module to remove common information and to reduce the differences in BUS, CDFI, and UE. Huang et al. [47] proposed a reinforcement learning-based auto-weighting strategy for the fusion of the BUS, CDFI, shear wave, and strain UE. Specifically, they provided a modality recovery method based on the modality feature consistency hypothesis and achieved a performance close to modality completeness when the ultrasound modalities were missing. Using BUS, CDFI, UE, and CEUS, Meng et al. [48] proposed a multistep modality fusion network and emphasized the mining of intermodal heterogeneity features by spatial–temporal feature collaboration and cooperation. Contrary to the results of the study by Xiang et al. [46] and Huang et al. [47], Meng et al. [48] included temporal and spatial information simultaneously and suggested that ultrasound modalities should provide features that could only be obtained by a certain modality. The performance of the method surpasses that of other related methods and provides a new framework for multimodal ultrasound fusion. In addition to clinical diagnosis, other interesting topics have also been discussed in some studies. Gao et al. [49] proposed a network for the tissue-aware diagnosis of cervical lymphadenopathy via dual-modal ultrasound semantic segmentation. The pixel-level localization of different tissue objects was realized first, and subsequent diagnosis was based on this region-division knowledge by integrating BUS and CDFI. The study [32] proposed a cross-modality lesion segmentation network for CEUS, where the lesion position could be determined under the guidance of BUS and the ROI could be delineated in a single CEUS frame image.

Finally, other imaging modalities, such as MRI, photoacoustic imaging, X-ray photoelectron spectroscopy, CT and magneto-acousto-electrical tomography, have been combined with ultrasound modalities [97–104]. These studies suggest that various imaging modalities have complementary advantages and can provide complementary functional information to improve the accuracy of disease diagnosis. Song et al. [104] implied that different imaging modalities could convey common information and that knowledge could be transferred from one modality to another, leading to the question of how to reasonably utilize information from different imaging modalities.

## Prospects

### Large-scale pre-trained models of ultrasound diagnosis

An important aspect of AI-based radiomics in ultrasound diagnosis is the development of large-scale pre-trained models. Pre-training refers to the process of training a model on a large dataset of images before fine-tuning it for a specific task. Pre-training enhances the model to learn generic features that can be used across different tasks and modalities, thereby improving the efficiency and effectiveness of the training. Recently, several large-scale pre-trained models have been developed for ultrasound diagnosis. These models have hundreds of millions of parameters and are trained on large-scale datasets of millions of images. These models can achieve state-of-the-art performance in various ultrasound diagnosis tasks, including image classification, segmentation, and detection. In addition, pre-trained models can be fine-tuned on small datasets to improve their performance in specific pathologies or patient populations.

Pre-trained models can assist clinicians in the interpretation of ultrasound images by providing automated diagnoses or recommendations, saving time and reducing errors. Furthermore, large-scale pre-trained models can improve access to healthcare. Ultrasound is a widely used diagnostic tool in both developed and developing countries. However, access to skilled operators and specialists may be limited, particularly in rural and remote areas. Pre-trained models can be deployed on portable ultrasound devices, enabling non-specialists to perform diagnostic tests with a high degree of accuracy. This can improve the availability of health care services in underserved communities and reduce the burden on specialist health care providers. In addition, large-scale pre-trained models have the potential to improve ultrasound diagnosis. Pre-trained models can be used to extract high-level features from large-scale ultrasound datasets. This can be employed to identify new biomarkers or diagnostic criteria for specific pathologies. It can lead to the development of new diagnostic tools and therapies to improve patient outcomes. In addition, pre-trained models can be used to evaluate the efficacy of new diagnostic or therapeutic interventions in clinical trials, which can improve the efficiency of drug development and regulatory approval.

### Artificial general intelligence models in ultrasound diagnosis

The ultimate prospect of AI-based radiomics for ultrasound diagnosis is the development of artificial general intelligence (AGI) models. This refers to AI models that can perform various clinical tasks and not focus solely on specific pathologies. Moreover, AGI models can learn from large-scale datasets and generalize to new tasks and modalities, thereby significantly improving the efficiency and effectiveness of clinical practice. Furthermore, AGI diagnostic models can provide versatility and adaptability, enabling them to identify abnormalities that are not explicitly labeled in the training data. The potential benefits of AGI models for ultrasound diagnosis are significant. These models can provide rapid and accurate diagnoses, which reduces the time and resources required for traditional diagnostic methods. They can also be used to predict patient outcomes and recommend personalized treatment plans based on individual patient characteristics.

Currently, all AI ultrasound diagnostic models are artificial narrow-intelligence models designed to construct and train models for specific clinical problems. These models have been successful in identifying specific pathologies (such as tumors or lesions); nonetheless, they lack the versatility and adaptability of AGI models. Although AGI models are still in their infancy, several promising developments have been achieved recently. For example, deep reinforcement learning has been used to develop AGI models that can perform a range of clinical tasks, including ultrasound diagnosis. Additionally, deep reinforcement learning enables a model to learn from feedback and adapt its behavior based on the context, which can improve its ability to perform complex and dynamic tasks. In ultrasound diagnosis, AGI can potentially be used to develop a system that can perform various diagnostic tasks such as identifying different types of lesions, classifying different stages of diseases, and predicting treatment outcomes. It can also be used to integrate data from multiple modalities such as ultrasound, MRI, and CT.

### Large-scale ultrasound dataset

The AGI diagnostic models can provide human-level intelligence in ultrasound diagnoses, thereby providing highly accurate diagnoses. However, the development of these models requires significant advancements in AI technology and the availability of high-quality ultrasound data. One of the main challenges in the development of AI-based radiomics models for ultrasound diagnosis is the lack of large-scale and diverse datasets. Contrary to other medical imaging modalities (such as CT and MRI) which have publicly available datasets with thousands of images, ultrasound datasets are usually small and limited in scope. This makes training robust and accurate AI models that can be generalized to different patient populations and pathologies difficult.

However, owing to the increasing popularity of ultrasound imaging and the growing availability of electronic health records, there is an opportunity to collect and curate large-scale ultrasound datasets. This facilitates the development of more accurate and robust AI models that can improve the diagnosis of various pathologies. In addition, large-scale datasets can facilitate the development of personalized medical approaches in which AI models can be trained to predict patient outcomes and guide treatment decisions based on individual characteristics.

The availability of large-scale ultrasound datasets is essential for the development of accurate and reliable AI models for ultrasound diagnosis. The development of ultrasound imaging technology has enabled the quick and efficient acquisition of large amounts of ultrasound data. However, the quality and consistency of the data can vary, and annotation or labeling may be limited. Efforts are underway to address these challenges by creating large-scale ultrasound datasets using standardized image acquisition protocols and high-quality annotations. For example, the Radiological Society of North America (RSNA) has launched the RSNA-ASNR AI Challenge, which provides a dataset of over 17000 ultrasound images of the carotid artery along with expert annotations. The availability of large-scale ultrasound datasets will enable the development of more robust AI models for ultrasound diagnosis and facilitate the validation and comparison of different AI models.

## Conclusions

Ultrasound diagnosis is widely used in clinical practice because of its noninvasive and real-time imaging capabilities. Radiomics, an emerging field in medical imaging, can extract quantitative features from medical images and provide a comprehensive analysis of image data. The application of AI in radiomics has significantly improved the accuracy and efficiency of ultrasound diagnosis. Static ultrasound images such as BUS, CDFI, and UE are widely used in clinical practice. Additionally, AI-based radiomics can analyze the texture, shape, and other quantitative features of images to identify patterns and provide diagnostic information. For example, AI-based radiomics have been used to differentiate benign from malignant thyroid nodules using BUS images with high accuracy. Dynamic ultrasound videos such as CEUS, provide additional information on the blood flow and perfusion of the imaged tissue. Additionally, AI-based radiomics can be used to analyze temporal changes in contrast enhancement and provide a more accurate diagnosis of liver tumors, breast lesions, and other diseases. Multi-modal ultrasound fusion analysis combines multiple ultrasound imaging modalities (such as BUS, CDFI, UE, and CEUS) to provide a comprehensive analysis of the imaged tissue. Furthermore, AI-based radiomics can be used to analyze the quantitative features of these images to provide a more accurate and comprehensive diagnosis. For example, AI-based radiomics has been utilized to differentiate malignant and benign breast lesions using a combination of BUS, CDFI, and CEUS images. In conclusion, AI-based radiomics has a considerable potential for improving the accuracy and efficiency of ultrasound diagnoses. By analyzing the quantitative features of static ultrasound images, dynamic ultrasound videos, and multi-modal ultrasound fusion, AI-based radiomics can provide a more accurate diagnosis of various diseases (including liver tumors, breast lesions, and thyroid nodules). However, some challenges (such as the need for large-scale datasets and standardized imaging protocols) must be addressed. With further research and development, AI-based radiomics are expected to play an increasingly important role in ultrasound diagnosis.

## Data Availability

Not applicable.

## Abbreviations

B-mode: Brightness-mode
AI: Artificial intelligence
CT: Computed tomography
MRI: Magnetic resonance imaging
BUS: B-mode ultrasound
CDFI: Color doppler flow imaging
UE: Ultrasound elastography
CEUS: Contrast-enhanced ultrasound
ROI: Region of interest
CNN: Convolutional neural network
DLRE: Deep learning radiomics of elastography
SWE: Shear wave elastography
HCC: Hepatocellular carcinoma
TACE: Transarterial chemoembolization
R-DLCEUS: Radiomics-based CEUS model
R-TIC: Radiomics-based time intensity curve of the CEUS model
R-BMode: Radiomics-based BUS image model
PFS: Progression-free survival
RFA: Radiofrequency ablation
SR: Surgical resection
IOU: Intersection over union
TB: Tumor boundary
AGI: Artificial general intelligence
DLR: Deep learning radiomics
SVM: Support vector machine
